# An Efficient Anomaly Recognition Framework Using an Attention Residual LSTM in Surveillance Videos

**DOI:** 10.3390/s21082811

**Published:** 2021-04-16

**Authors:** Waseem Ullah, Amin Ullah, Tanveer Hussain, Zulfiqar Ahmad Khan, Sung Wook Baik

**Affiliations:** Sejong University, Seoul 143-747, Korea; waseem@sju.ac.kr (W.U.); qamin3797@sju.ac.kr (A.U.); tanveer@sju.ac.kr (T.H.); zulfiqar@sju.ac.kr (Z.A.K.)

**Keywords:** anomaly detection, video surveillance system, abnormal activity recognition, attention mechanism, LSTM, residual LSTM, deep learning, smart surveillance, crime recognition

## Abstract

Video anomaly recognition in smart cities is an important computer vision task that plays a vital role in smart surveillance and public safety but is challenging due to its diverse, complex, and infrequent occurrence in real-time surveillance environments. Various deep learning models use significant amounts of training data without generalization abilities and with huge time complexity. To overcome these problems, in the current work, we present an efficient light-weight convolutional neural network (CNN)-based anomaly recognition framework that is functional in a surveillance environment with reduced time complexity. We extract spatial CNN features from a series of video frames and feed them to the proposed residual attention-based long short-term memory (LSTM) network, which can precisely recognize anomalous activity in surveillance videos. The representative CNN features with the residual blocks concept in LSTM for sequence learning prove to be effective for anomaly detection and recognition, validating our model’s effective usage in smart cities video surveillance. Extensive experiments on the real-world benchmark UCF-Crime dataset validate the effectiveness of the proposed model within complex surveillance environments and demonstrate that our proposed model outperforms state-of-the-art models with a 1.77%, 0.76%, and 8.62% increase in accuracy on the UCF-Crime, UMN and Avenue datasets, respectively.

## 1. Introduction

In the 21st century, one of the leading causes of lost lives and property is the surge in the crime rate, as compared to other issues [1]. An intelligent video surveillance system is a most preferred solution for the quick and early detection of such unusual events. Anomalous event recognition in surveillance videos demands much attention due to its vast applications in many domains, including crime prevention, automated intelligent visual monitoring, and traffic security [2]. To avoid any mishap and ensure public safety, for the past few decades, a vast amount of surveillance cameras have been deployed in private and public places for effective real-time monitoring. However, most of these cameras provide only passive recording services and lack monitoring capabilities. The volume of these videos increases each minute, making understanding and analyzing them effortful for human experts. Similarly, surveillance analysts must wait for hours to capture or witness anomalous events for instant reporting. Due to the rareness of real-world anomalous events, video anomaly recognition has previously been investigated as a one-class classification problem [3,4,5], i.e., the model is trained on normal videos, and in the test set, a video is classified as anomalous when abnormal patterns are encountered. It is not feasible to accumulate all the usual events of real-world surveillance in a single dataset. Hence, various normal behaviors might stray from normal events in the training set and eventually generate false alarms.

Recognizing anomalies in surveillance videos is an extremely hard and challenging task, for reasons including a subjective definition of anomaly, an inadequate amount of annotated data due to the infrequent occurrence of anomalous events, the lower resolution of surveillance videos, and the large number of intra/inter class variations. In the literature, various stochastic and discriminative techniques using low-level features are proposed [6,7]. However, the performance of these techniques is influenced by the hyper-parameter settings. Unfortunately, it is impractical and problematic to get the optimal solution for all the given videos. As a result, the demand for these methods declined, replaced by demand for various deep learning-based methods [8,9,10] due to their sophisticated performance. In recent years, various models were proposed using both usual and unusual events to learn anomalous patterns [11,12,13]: these models are trained on both usual and unusual videos. Frame level annotation is a considerably expensive and time-consuming process; therefore, to overcome this limitation, weakly supervised learning techniques for video level labels are generally applied in anomalous event detection [11,12,13].

For the past two decades, a massive amount of research has been carried out on video abnormal event recognition [14,15,16,17]. Depending on whether the labels of the classes are available or not, the baseline anomalous event detection techniques in real-world environments can be classified into the subsequent three policies. The preliminary policy is a supervised approach, which involves usual and unusual events equally; these methods learn predictive patterns for both usual and unusual events and check which model is best suited to the given data. However, this policy is impractical, as the unusual events are unbounded and unexpectable in the surveillance environment, and it is almost unworkable to accumulate all of the types of unusual events. Furthermore, the supervised-based techniques can only be used on specific regions/scenes since they are applying previous knowledge and basic data to design inadequate distributions. Therefore, the attention of mainstream research is diverted towards the second strategy, which is semi-supervised learning [18,19]. The third policy is the unsupervised approach, which checks for earlier undetected patterns in the raw video without appropriate labels and requires minimum human supervision. These types of techniques detect abnormal events mostly by the characteristics and properties of the data [6,20]. The main drawback of these techniques is that they neglect the global information that needs to be considered in future research.

Existing deep feature-based models comprise autoencoders and ranking-based techniques, accomplishing quite reasonable results but produce higher false alarms and the accuracy of these techniques is not promising for recognizing abnormal activities. Consequently, the performance of most of the existing techniques for anomaly detection is inadequate for handling complex surveillance environments. To overcome these problems, in the current work, we introduce a light-weight model to recognize anomalies in real-world surveillance videos. Our model learns visual features from a sequence of consecutive video frames of incorporating them in the spatiotemporal information for surveillance videos. The summarized key contributions of our work follow:

- We propose a light-weight model for anomaly detection, functional for a real-world surveillance network. We adopted a pretrained model and extracted frame-wise features, followed by a sequential learning mechanism for the precise recognition of anomalous activity.
- We employed the residual attention-based long short-term memory (LSTM) concept, which can effectively learn temporal context information and precisely recognize anomalous activity. Moreover, using a residual attention-based LSTM saves more than 10% of learnable parameters as compared to the usual LSTM network size.
- Our proposed model is tested using the challenging University of Central Florida UCF-Crime dataset, outperforming the baseline methods in terms of accuracy with reduced number of model parameters and size compared to existing anomaly activity recognition models.

The rest of this manuscript is structured as follows: Section 2 gives a brief overview of the existing techniques of anomaly detection and recognition in the literature. Section 3 is a detailed explanation about the materials and methods that are used for abnormal activity recognition. The model implementation and experimental results, along with the evaluation of the proposed model are discussed in Section 4, followed by the conclusion of the current work in Section 5.

## 2. Related Work

The anomaly detection and recognition problems in the surveillance environment are extensively studied in the existing literature. In the current section, the existing anomaly detection techniques are summarized in two broad categories; traditional feature-based techniques and deep learning-based techniques for anomalous event recognition are discussed in detail in subsequent sections.

### 2.1. Traditional Feature-Based Techniques

Previously, low-level feature-based methods were extensively applied for anomaly detection. These methods are mainly based on three phases: (1) feature extraction, where the low-level pattens are extracted from the training set; (2) learning from the features to distinguish the distribution of encoding regular patterns or normal events; and (3) identifying the outliers or isolated clusters as anomalous events. For the feature extraction phase, earlier approaches mostly employ low-level trajectories, image coordinates, and regular patterns [21,22]. However, these techniques do not perfectly provide appropriate performances in crowded or complex occurrences with multiple shadows and occlusions, as trajectory-based features mostly fail in such cases. To handle the problems of the trajectory features, the researchers introduced the alternative feature procedures known as low-level spatiotemporal features, including a histogram of oriented gradients and a histogram of oriented flow, broadly utilized for anomaly detection [23,24]. Taking advantage of spatiotemporal features, Zhang et al. [25] used the Markov random field for modeling the usual events. Kim and Grauman [26] proposed a system that used a Markov random field model to detect unusual events in videos. To learn the normal patterns of each event at a local node, they captured the distribution of the continual optical flow observation and atomic motion patterns using a mixture of probabilistic principal component analyzers. Another study proposed detecting the frequently occurring local histograms by an exponential distribution of the optical flow [27]. The authors in [28] proposed a Gaussian mixture model-based technique to integrate dynamic textures. Dictionary learning and sparse coding is a famous technique used to encode normal patterns and detect abnormal events [6,29,30]. The core idea of these techniques is that the usual patterns are characterized on the basis of a dictionary, which is used to encode the normal patterns in the training set. Consequently, the patterns are considered usual/normal when their reconstruction error is low, while the pattern is seen as abnormal/unusual when its reconstruction error is high. The main drawback of these methods is that optimizing the sparse coefficients is generally time consuming.

### 2.2. Deep Feature-Based Techniques

In the current era, deep feature-based models have achieved great success in numerous domains of nonlinear high dimensional data, such as activity recognition [31] and video summarization [32], among many others [33,34]. Most of the previous literature is based on semi-supervised anomaly detection techniques in which the model is trained on normal data. Liu et al. [18] proposed a framework that used a convolutional neural network (CNN) as an encoder to encode the video frames, and ConvLSTM was applied to detect anomalous events. Their encoder efficiently encodes the changes in motion for detecting anomalies in a surveillance environment. Similarly, Parab et al. [35] introduced a system based on a CNN and LSTM to detect unusual situations at an automated teller machine. In this model, the frame-level features are extracted from the videos and then fed to a bidirectional LSTM to classify abnormal events at an automated teller machine. In our pioneering work, we used deep CNN features from a series of frames and passed them through a multilayer bidirectional LSTM to learn the spatiotemporal information of the input video and detect abnormal events [36]. Luo et al. [18] suggested a convolutional LSTM with an autoencoder-based model for anomaly detection in videos. Additionally, they extended his work using a stacked recurrent neural network (RNN) with an autoencoder to detect anomalies. Hasan et al. [37] recommended a system for anomaly detection established on a convolutional autoencoder, followed by a RNN. Liu et al. [14] introduced a system in which the fusion of a temporal and spatial detector is presented to detect anomalies in videos. In this model, the discriminant saliency detector and a set of dynamic texture features are modeled as normal events from the training data. Liu et al. [14] introduced a model for future frame prediction for anomaly detection that prevents the identity mapping and also increases its functioning in anomaly detection. Additionally, generative models are one of the popular techniques that are utilized to detect abnormalities in videos. Sabokroul et al. [38] suggested generative adversarial networks (GANs) to detect anomalies in the surveillance environment. In this model, they use GANs with discriminator and generator methods to learn the normal distribution. Deng et al. [39] introduced a model called the “Spatio-Temporal Autoencoder”, where they applied a deep neural network to extract both temporal and spatial features from videos. Furthermore, they introduced weight-reducing projection loss to predict future frames effectively and learn motion features in videos. Cheng et al. [40] introduced a clustering-based deep autoencoder to produce efficient information within usual events. Spatiotemporal feature regularity is learned using two modules. In the first module, the spatial autoencoder manages the last individual video frame, and the second module is a temporal autoencoder, which operates and constructs the RGB difference from the rest of the frames. To detect anomalies in videos, supervised learning-based techniques have been well studied over the past few years. Recently, weakly supervised-based state-of-the-art techniques for video labeling have been recommended in studies [12,41], where the detection of anomalous events is performed using C3D [42] and multi-instance learning (MIL) [43,44]. Sultani et al. [12] proposed a framework based on weak video labels using deep features and the MIL approach to detect anomalous events. This paradigm is trained on both normal and abnormal videos by generating two different bags for usual and unusual events, and the MIL method was applied to detect anomalous event scores in the videos. Tan et al. [45] introduced an anomaly detection technique which efficiently used sparse components and hyperspectral image pixel decomposition into lower ranks. Furthermore, they used a spatial constraint for lower ranks, which uses a single or multiple local window technique to represent and smooth the coefficients for effective anomaly detection. Following the ranked-based technique [46], an abnormal event detection framework was introduced by using MIL with a graph-based technique to represent the normal and abnormal events. Zhu et al. [13] proposed a temporal augmented network with MIL by incorporating an attention block and achieved state-of-the-art performance for normal and abnormal event detection. Kuldeep et al. [47] suggested a system known as “DEARESt” anomaly recognition. This system is based on two flow feature networks: one uses CNN-based features while the other uses motion features separately. In our pioneering work, we used deep CNN features from the series of frames and passed them through a multilayer bidirectional LSTM to learn the spatiotemporal information of the input video and detect the abnormal events [36].

## 3. Materials and Methods

**Figure 1:**
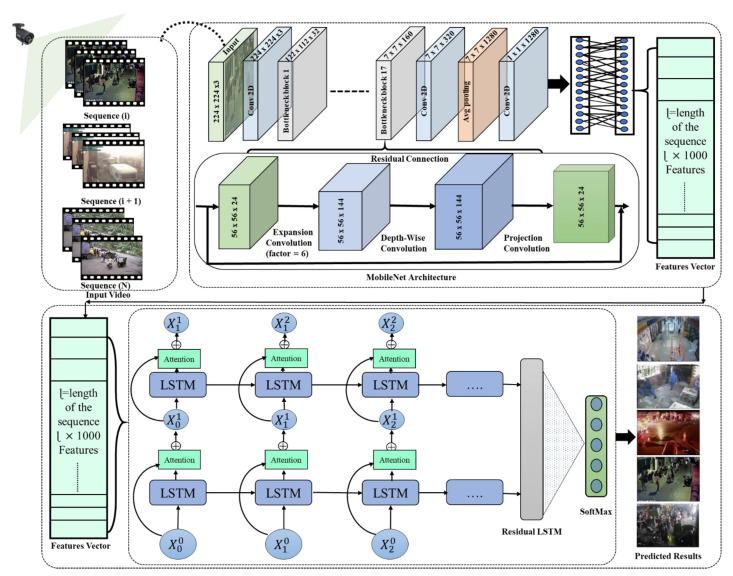
The proposed framework for anomaly recognition in the surveillance environment.

In this section, we discuss the overall structure of our proposed model and its key elements which are presented in Figure 1. The proposed system is divided into three key phases: the surveillance video frames are passed from the pretrained light-weight CNN model to extract features; we generate a feature vector from a series of 30 frames of the video; and this feature vector is fed to the residual LSTM to recognize anomalous activities in a real-world environment. Each phase of the model is discussed in detail in subsequent sections.

### 3.1. Feature Extraction Using Light-Weight CNN

**Figure 2:**
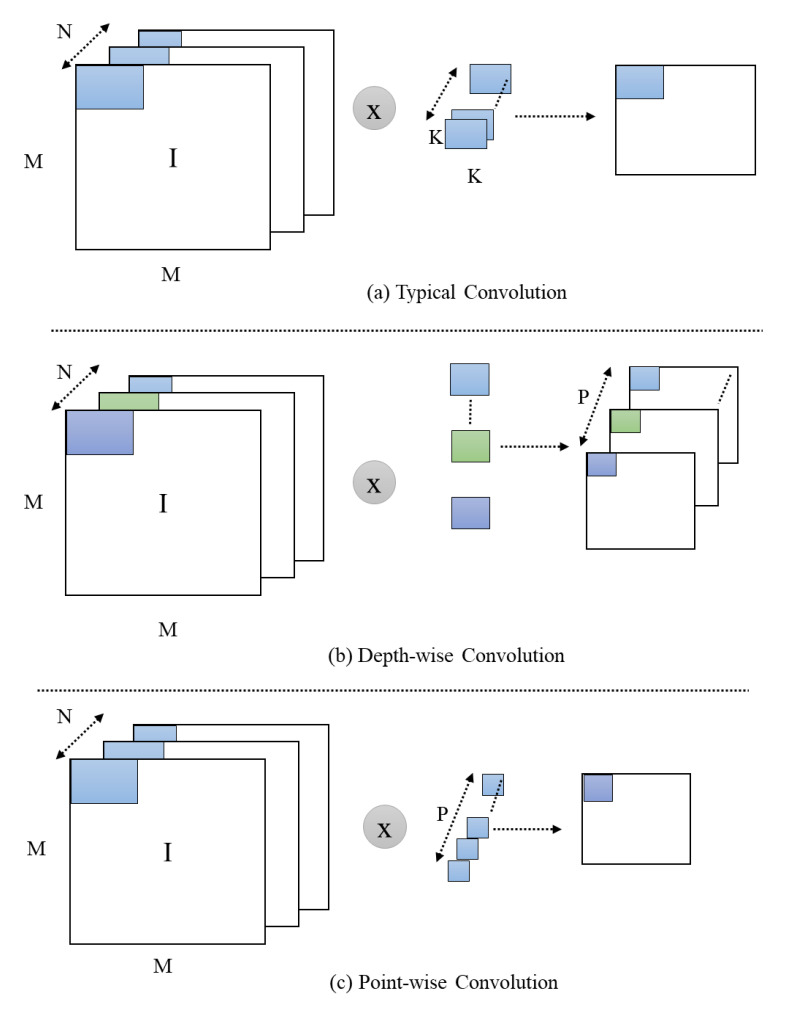
Representation of typical, depth-wise, and pointwise convolution, where I, M, and N represent image, dimensions, and number of channels, respectively.

The core concept at backend of MobileNet paradigms is to supplant huge and costly convolutional layers with depth-wise distinguishable convolutional blocks. These convolutional blocks consist of two key elements: (1) a depth-wise convolutional layer that uses 3 × 3 filters for a given input, and (2) point-wise convolutional layers that incorporate a 1 × 1 filter that functions to merge these filtered values and extract the learned features. The MobileNet model is light-weight and considerably faster than a conventional convolution-based model and achieves approximately the same results. The MobileNetV1 has 3 × 3 convolution and 13 depth-wise distinguishable convolutional blocks [48]. MobileNetV2 contains one extra expansion layer in each block with a filter size of 1 × 1 for point-wise and depth-wise convolutional layers [49]. The core objective of this layer is to increase the amount of channels in the data rather than moving to the depth-wise convolution. As a result, this layer generates more output channels than the given input channels. Unlike V1, the point-wise layers of MobileNetV2 are part of the projection layer: this layer is responsible for projecting data with huge amount of channels into tensors, along with a small amount of channels (Figure 2). The main function of the bottleneck residual block is that it provides the end result of these blocks; the residual block is modified, using convolutions to build a bottleneck. This block is valuable for reducing the number of parameters and matrix multiplications. As usual, every layer of MobileNetV2 contains ReLU6 and batch normalization, which is used as an activation function. The results of the projection layer are generated without using the activation function, but the overall composition of the MobileNet involves 17 bottleneck residual blocks and regular 1 × 1 convolution, followed by a global average pooling and classification layer. The top of MobileNetV2 is the global average pooling layer, which is helpful in reducing the problem of overfitting. The MobileNetV2 model is pretrained on the challenging ImageNet dataset, which consists of 1000 classes and approximately 1.4 M images, and we use these weights in our model. We exclude the topmost layer of the MobileNetV2, which is ideal for feature extraction. We extracted features from a 30-frame sequence; these features are further passed through our proposed residual attention-based LSTM to recognize anomalous activities. 

### 3.2. Sequential Learning Techniques

LSTM was introduced to resolve the vanishing or exploding gradients issue in recurrent neural networks, and involves internal memory cells that are controlled by an input and forget gate network. The cell state is altered by the forget gate ranked under the cell state and also modified by the input gate. Additionally, the main purpose of the forget gate in the LSTM is to decide how much information from the previous memory should be passed into the next time step. Similarly, the input gate first regulates how much new information should be entered into the memory cell and then a vector is formed applying the tanh function, which provides output. Depending on these gates, LSTM can handle the short- and long-term dependency of the sequential information [50]. The LSTM formulation is expressed as follows:

(1) $$C_{t}^{s}=\tan h\left(ŵ{ɧ}_{g}*{ɧ}_{t-1}^{s}+\;{ŵ}_{xg}^{s}*x_{t}^{s}\right)$$

(2) $$ʄ{\;}_{t}^{s}=\sigma \left(ŵ{ɧ}_{ʄ}*{ɧ}_{t-1}^{s}+\;{ŵ}_{xʄ}^{s}*x_{t}^{s}\right)$$

(3) $${į\;}_{t}^{s}=\sigma \left(ŵ{ɧ}_{į}*{ɧ}_{t-1}^{s}+\;{ŵ}_{xį}^{s}*x_{t}^{s}\right)$$

(4) $${Ō\;}_{t}^{s}=\sigma \left(ŵ{ɧ}_{Ō}*{ɧ}_{t-1}^{s}+\;{ŵ}_{xŌ}^{s}*x_{t}^{s}\right)$$

(5) $$C_{t}^{s}=ʄ{\;}_{t}^{s}{ʘ}_{t-1}^{s}+\;{į\;}_{t}^{s}\;ʘ\;C_{t}^{s}$$

(6) $${ɧ}_{t}^{s}={Ō\;}_{t}^{s}\;ʘ\;\tan h\left(C_{t}^{s}\right)$$

In the above equations, the weights ${ŵ}_{ɧ*}$ throughout the parallel LSTM architecture, including the $ŵ{ɧ}_{g}$,$\;ŵ{ɧ}_{ʄ}$ ,$\;ŵ{ɧ}_{į}$, and $ŵ{ɧ}_{Ō}$ parameters are employed to control and monitor the prior time step’s information concerning the hidden states along with ${ŵ}_{xg}$,$\;{ŵ}_{xʄ},\;{ŵ}_{xį}$, and ${ŵ}_{xŌį}\;$, which are applied for the current input time steps’ weight matrices. The superscript s indicates the input sequence information, the subscript t displays the time step information, σ is used for the sigmoid function, and ʘ represents elementwise multiplication.

### 3.3. Residual Attention-Based LSTM

A residual learning technique was proposed for image recognition to train ultra-deep CNNs [51,52]. The residual concept is employed to characterize the top-level layers’ sequential information and the reformulation of layers by discovering residual functions, given as the input layer [53]. Generally, the residual function learning can be formulated as follows:

(7) Y=  ʄ(Ẍ ,Ẅ)+Ẍ

**Figure 3:**
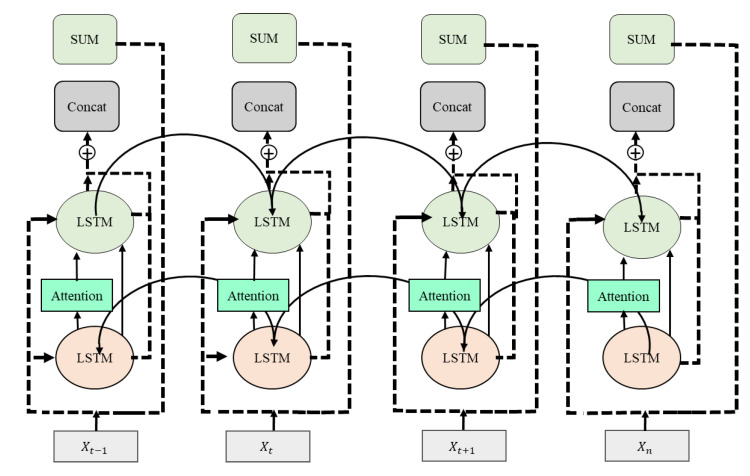
The residual long short-term memory (LSTM) architecture.

In Equation (7), the given input and resultant sequential information vectors of the layers are considered Ẍ and Y. The ʄ (Ẍ ,Ẅ) demonstrates the residual learned from the related layers. The results of these layers in the residual learning, which develops a sequence of the given input and nonlinear residual, are presented in Figure 3. The main advantage of this technique is that it creates a shortcut function among the several layers for more effective training of the model, and it is also useful for preventing the main issue of vanishing gradients owing to the composition with the adapting residual ʄ (Ẍ ,Ẅ).

In this study, we normalize the information by employing the normalization layer [54] in a residual LSTM to ease the dynamic hidden state, normalize the information of the neurons for the LSTM and also reduce the training time of a deep RNN as follows:

(8) $${ñ}_{t}=\frac{1}{𝕙}\;\sum_{i=0}^{𝕙}\left(ɧt\right)i$$

(9) $${ẟ}_{t}=\sqrt{\frac{1}{𝕙}\;\sum_{i=0}^{𝕙}{\left(\left(ɧt\right)i-{ñ}_{t}\right)}^{2}}$$

(10) $${ỳ}_{t}=\;ʄ\;\left(\frac{ġ}{{ẟ}_{t}}\;ʘ\;\left(ɧt-{ñ}_{t}\right)+b\right)$$

where (ɧt)i is the hidden state in each layer of the LSTM of the ἰth neuron, ġ and b are the trainable weights that are used to rescale the input sequence of the activation function ʄ, and the time step is represented using the subscript t. We applied a dropout threshold of 0.5 in each layer of the residual LSTM before the forward connections to reduce overfitting. A baseline research [55] used encoder and decoder with attention mechanisms to enhance the performance of their video captioning model. They employed decoder for word generation that inputs video features corresponding to their next word, which is based on the words previously produced by the model. This technique effectively generates video captions using two type of inputs, including natural language processing 1D feature vector and video frames 2D data. Inspired by [55], we also used self-attention layer with residual LSTM, that functions for both short- and long-term dependencies by utilizing latent correlation among features at various positions. This self-attention layer produces context-aware vector and temporal order representation for sequential features. In contrast to the video captioning model, in our case, we have only one input, which is feature vector from the video frames sequence that we input to residual attention-based LSTM that requires a single block of features for sequence learning.

(11) $$k_{t}=\frac{1}{𝕙}\sum_{i=1}^{𝕙}ŵ{\;}_{t}^{i}{ʄ}_{i}$$

(12) $$\dot{{Տ}_{t}}={ŵ}^{T}\tan h\left({ŵ}_{ɧ}\;ɧt+M_{ɧ}R_{f}+b_{ɧ}\right)$$

(13) $${Ą}_{t}=softmax\left({Տ}_{t}\right)$$

In the above equations,$\;{ŵ}^{T},{ŵ}_{ɧ}\;,M_{ɧ},\;b_{ɧ}\;$are the parameters learned for the frame features ${ʄ}_{i}$ according to the attention weight $ŵ{\;}_{t}^{i}$ to return the score ${Տ}_{t}$. Finally, ${Ą}_{t}$ shows the output probabilities attained from the Softmax classification layer. The extracted deep features of the 30-frame sequence are used to recognize that the sequence contains either abnormal activities or normal events, which are passed from the residual attention-based LSTM, and final predictions are performed using the SoftMax layer. Several experiments were performed to select the best hyperparameter settings, and finally we choose Adam as an optimizer, with a learning rate of 0.01, and categorical cross-entropy as a loss function. The batch size was 32 and the number of epochs was 200 for training the model. We stopped the training process when the loss no longer decreased.

## 4. Results 

We experimentally assessed our proposed model using the benchmark anomaly detection UCF-Crime dataset [56]. To test the performance of the proposed paradigm, we experimentally evaluated it across numerous metrics, including the confusion matrix, F1 score, recall, precision, class-wise accuracy, area under curve (AUC), and receiver operating characteristic (ROC) curve. The performance of our model is compared with recent abnormal activity recognition techniques. The proposed model is implemented using Keras and backend TensorFlow with Python 3.6 on a Windows 10 platform and Corei5-6600 setup with 16-GB RAM, equipped with a 12-GB GeForce-Titan-X graphics processing unit (GPU).

### 4.1. Datasets

In this work, the performance of the proposed model is extensively evaluated on various benchmark datasets, i.e., the University of Minnesota UMN dataset [57], Avenue dataset [58], and UCF-Crime dataset [56]. The UCF-Crime dataset consists of 1900 long untrimmed videos for 13 real-world anomalous events including fighting, stealing, shooting, shoplifting, robbery, road accident, arson, abuse, arrest, assault, burglary, vandalism and explosion. The UCF-Crime dataset is an almost balanced dataset that contains 800 normal and 810 anomalous event videos in the training set. The rest of the videos of the dataset include 150 normal and 140 anomalous events that are temporally annotated to test the performance of the model. The challenging part of the UCF-Crime dataset is that it only contains temporal annotation for the testing set. We follow a former research strategy [47] to determine the training, testing, and validation ratio. The UMN dataset consists of 11 video sequences of various scenes of abnormal activities and is an extensively utilized dataset. This dataset has in total 4144, 2144, and 1453 frames of three scenes, plaza, indoor, and lawn, respectively. The Avenue dataset consists of 16 training and 21 testing videos and contains in total 30,652 frames. This dataset has 47 abnormal events, and the resolution of each frame is 360 × 640 pixels. 

### 4.2. Evaluation Methods

In this portion, to measure the performance and effectiveness of the proposed model, we used evaluation parameters often used for abnormal activity detection [6,12,59], such as the AUC and the receiver operating characteristic curve. We also evaluate our proposed model using the recall, F1 score, and precision. We applied these evaluation parameters on test videos and counted the total number of false negative (FN), true positive (TP), and false positive (FP) results.

### 4.3. Results

**Figure 4:**
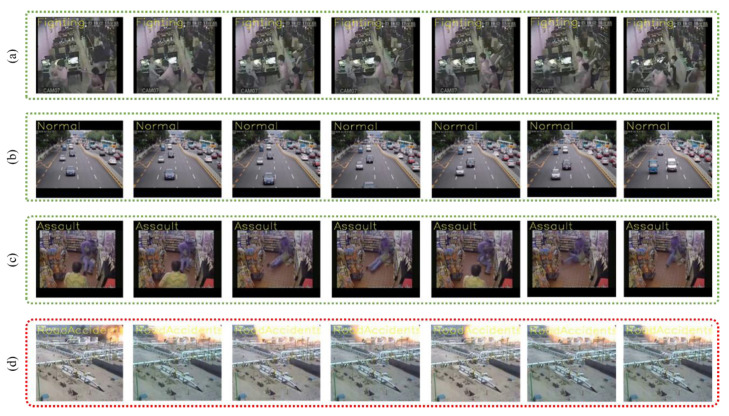
Visual results of the proposed model for anomaly activity recognition on test videos, where (**a**–**c**) are the correctly and (**d**) shows incorrectly predicted results; (**a**–**d**) show the prediction results on the fighting, normal, assault, and explosion categories, respectively; (**d**) has failure cases, where the model has predicted that an explosion is a road accident.

**Figure 5:**
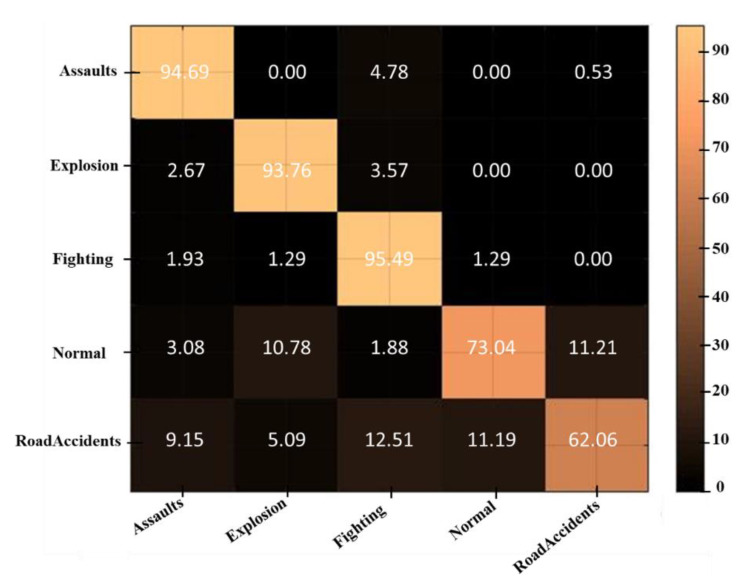
The confusion matrix of UCF-Crime dataset for anomalous activity recognition using residual LSTM.

**Figure 6:**
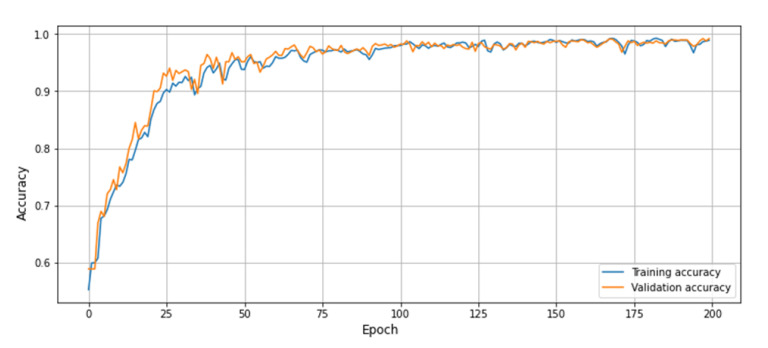
The training graph of the proposed model using UCF-Crime dataset.

**Figure 7:**
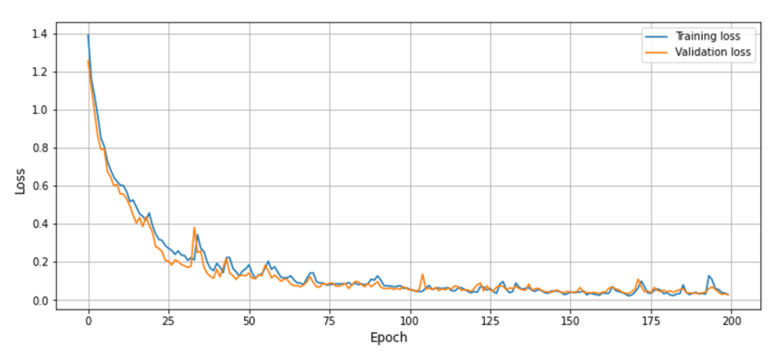
Training and validation loss graph of the proposed model using UCF-Crime dataset.

**Figure 8:**
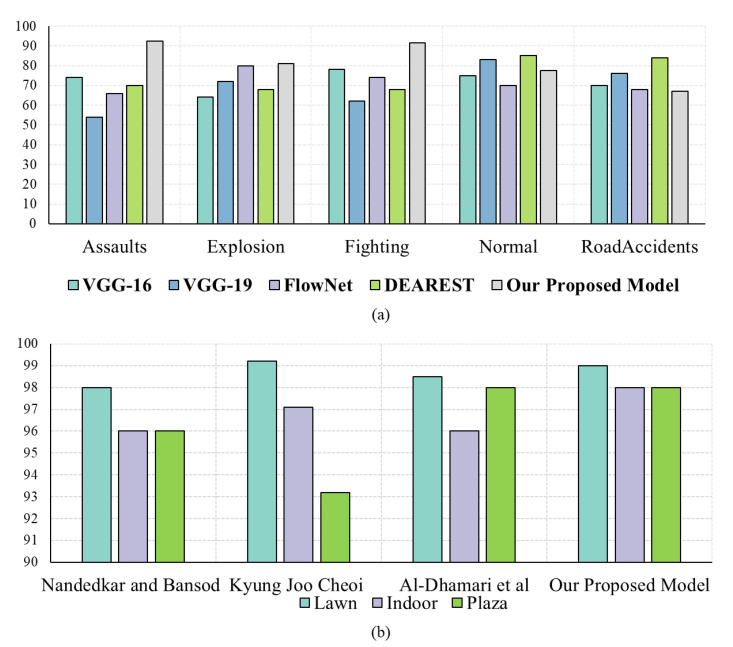
Comparison of class-wise accuracy for the (**a**) UCF-Crime and (**b**) UMN datasets of the proposed method with existing methods.

**Figure 9:**
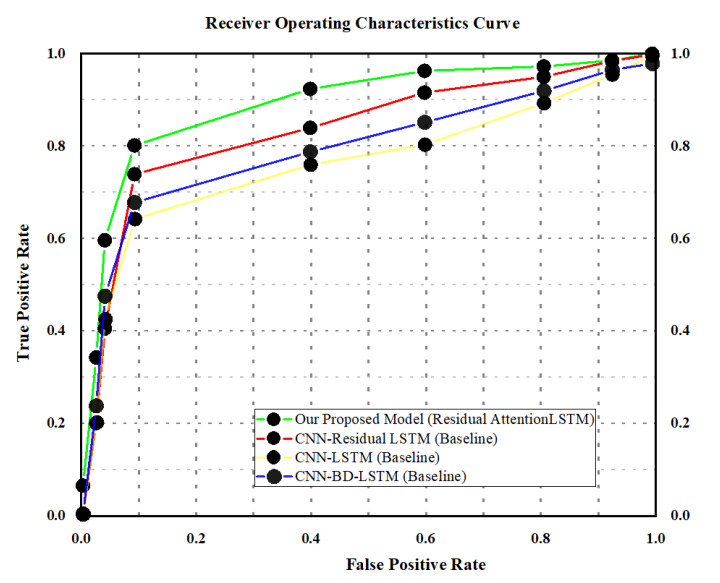
Comparative analysis of the proposed model with CNN-LSTM and CNN-BD-LSTM using ROC and AUC curves.

**Figure 10:**
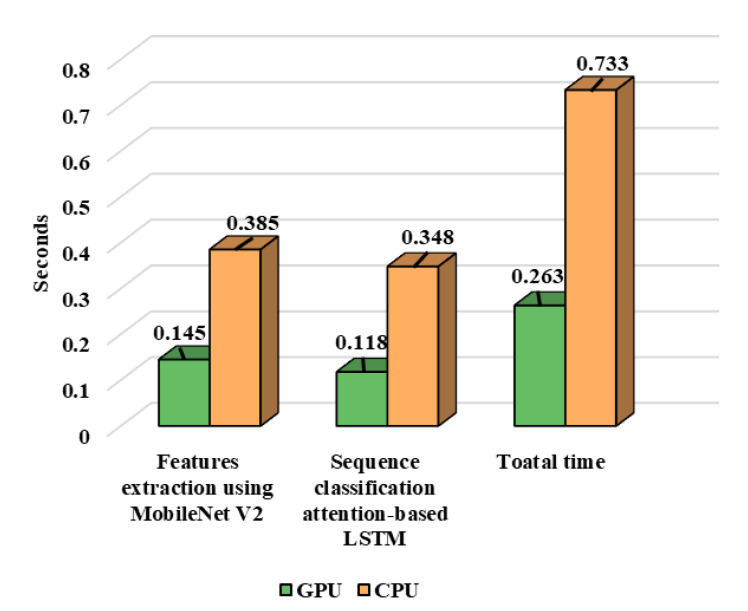
Performance analysis of proposed model using graphics processing unit (GPU) and central processing unit (CPU) for a 30 frames sequence.

In this portion, broad experiments are carried out to test the performance of our proposed model utilizing the UCF-Crime dataset. We perform experiments using the LSTM, bidirectional LSTM (BD-LSTM), and residual LSTM. We tried several variants of the LSTM in our experimental analysis before reaching the final choice of the proposed residual attention-based LSTM model, which has shown tremendous performance for the investigated problem of anomaly recognition. The proposed residual attention-based LSTM model achieved quite promising results as compared with baseline techniques, which are presented in Table 1. Some of the visual results of our proposed model for anomalous activity recognition are shown in Figure 4. Figure 4a–c show the accurate prediction results of the proposed model, and Figure 4d shows the incorrect prediction results. The prediction results of the UCF-Crime dataset of the residual LSTM are as follows: for each class, results are represented as a confusion matrix, provided in Figure 5. The training graph and loss and the class-wise accuracy of the proposed model are shown in Figure 6, Figure 7 and Figure 8, and the precision, F1 score, and recall are demonstrated in Table 1. The ROC and AUC curves of the proposed model are displayed in Figure 9. We calculated the time complexities of MobileNetV2, an attention-based LSTM, and our overall proposed model (Figure 10), and the floating point operations per second (FLOPS) of these models, which are converted from Giga FLOPs to Mega FLOPs, are 3.1, 615, and 618.1 (Table 2). 

### 4.4. Comparison with the State-of-the-Art Techniques

In this section, the performance of our proposed anomaly recognition model is compared with state-of-the-art techniques by using the UCF-Crime dataset. The authors of [47] checked various deep learning models, i.e., VGG-16, VGG-19, FlowNet, and DEARESt, respectively. The DEARESt model provides the best performance among these methods. In the modern era, deep learning models are becoming deeper and deeper, and also require huge amounts of storage; they also have increased computational complexity and stringent installation protocols over the edge node. In anomaly recognition, a delay in response can cost human lives and property; therefore, efficient model selection is a very important aspect for any anomaly recognition system. Our decision to use the light-weight CNN model MobileNetV2 is due to its small storage size, a smaller number of learned parameters, and its fast processing time, with a performance equivalent to heavy-weight CNN models [12,47,60]. The efficiency of the proposed model is compared with these existing techniques in terms of model size, time complexity, and the number of parameters, as shown in Table 2. We achieved overall accuracies of 78.43%, 98.20%, and 98.80%, which is increased by 1.77%, 0.76%, and 8.62% when compared to existing state-of-the-art techniques, with fewer parameters and a reduced model, as shown in Table 3. The proposed model can process a sequence of 30 frames in 0.263 s, which is comparatively lower than the recent existing techniques [47,62,63]. The sizes of existing models are much bigger, and their recognition performance is relatively low as compared to our proposed model, as shown in Table 2.

### 4.5. Discussion 

The main objective of this paper is to utilize a light-weight CNN model to detect and recognize anomalies efficiently. For many computer vision applications, CNN models are becoming deeper and deeper, making their applicability over edge devices questionable. To overcome these problems and achieve light-weight functionality, we utilize MobileNetV2 for feature extraction, followed by a residual attention-based LSTM for anomalous sequence recognition. MobileNetV2 is used to extract efficient features from input videos, improving the performance of the proposed attention-based LSTM. We use standard training and testing sets provided in the existing literature to test and compare the performance of the proposed model against rivals. Additionally, we tried several variants of LSTM in our experimental analysis, such as LSTM, BD-LSTM, and residual LSTM, before reaching our final decision to use the proposed residual LSTM model, which has shown tremendous performance for the investigated problem of anomaly recognition, as reported in Table 1. Moreover, the efficiency comparison of the proposed model in terms of time complexity, model size, and parameters utilization showed that it outclasses the existing models, as shown in Table 2. Table 3 shows the performance comparison of the proposed model with recent state-of-the-art techniques [47,60,61,62,63,64] using various benchmark datasets in terms of accuracy. The proposed model outperformed the existing techniques by increasing the accuracy by 1.77%, 0.76%, and 8.62% margins for the UCF-Crime, UMN, and Avenue datasets, respectively.

## 5. Conclusions

Smart surveillance systems are gaining attention among computer vision experts; they are mainly deployed for monitoring purposes. However, deep models are data-thirsty and demand heavy processing systems for effective analysis. In contrast, surveillance systems require quick countermeasures and responses against any abnormal events, detected automatically using computer vision systems. In the current study, we introduced a light-weight efficient model to recognize anomalies in smart cities with state-of-the-art accuracy by utilizing various challenging benchmark datasets. Our proposed model extracts deep CNN spatial features from a sequence of frames; then, it uses a residual attention-based LSTM to recognize anomalous events in a surveillance system. The usage of light-weight CNN features with a residual attention-based LSTM provides a high-level adaptability to smart surveillance environments. We validated our proposed model using various evaluation parameters. The proposed model is proven to have a higher accuracy than existing anomaly recognition methods. The experimental results of our proposed model reveal better accuracies with increase of 1.77%, 0.76%, and 8.62% in accuracy for the UCF-Crime, UMN, and Avenue datasets, respectively, and considerable improvements in reducing false alarm rates compared to the abnormal activity recognition literature.

In future works, we aim to investigate other deep learning models, 3D models, graph neural networks, and multi-instance learning formulations to enhance the system performance. Additionally, we intend to develop a generative technique appropriate for recognizing more classes of anomalies. 

## Figures and Tables

**Table 1:** Performance evaluation of the proposed deep models using convolutional neural network (CNN) features and best results are represented in bold.

Model	Dataset	Recall (%)	Precision (%)	F1 Score (%)	AUC (%)
Mobile Net V2 +LSTM	**UCF-Crime dataset**	86	74	77	88
Mobile Net V2 +BD-LSTM		79	84	76	87
Mobile Net V2 + residual LSTM		91	78	82	95
Our Proposed Model		**78**	**87**	**81**	**96**
Mobile Net V2 +LSTM	**UMN**	87	77	81	86
Mobile Net V2 +BD-LSTM		88	81	84	88
Mobile Net V2 + residual LSTM		94	95	94	96
Our Proposed Model		**98**	**98**	**98**	**98**
Mobile Net V2 +LSTM	**Avenue**	91	93	92	91
Mobile Net V2 +BD-LSTM		94	95	94	94
Mobile Net V2 + residual LSTM		93	94	94	94
Our Proposed Model		**98**	**99**	**99**	**98**

**Table 2:** Efficiency comparison of the proposed model with state-of-the-art techniques in terms of model size, parameters, FLOPS, and time complexity using UCF-Crime dataset and best results are represented in bold.

Model	Time Complexity (Seconds)	Model Size (MB)	Parameters (Millions)	FLOPs (Mega)
VGG-16 (2014) [60]	-	528	138	
VGG-19 (2014) [60]	-	549	143	
FlowNet (2017) [61]	-	638.5	162.49	
DEARESt (2018) [47]	-	1187.5	305.49	
Our Proposed Model	**0.263**	**12.8**	**3.3**	**618.3**

**Table 3:** Performance of the proposed model with state-of-the-art techniques and the best results are represented in bold.

Model	Accuracy (%)		
	UCF-Crime [56]	UMN [57]	Avenue [58]
VGG-16 (2014)[60]	72.66	-	-
VGG-19 (2014)[60]	71.66	-	-
FlowNet (2017)[61]	71.33	-	-
DEARESt (2018)[47]	76.66	-	-
Nandedkar and Bansod (2019)[62]	-	96.99	-
Kyung Joo Cheoi (2020)[64]	-	96.50	90.18
Al-Dhamari et al. (2020) [63]	-	97.44	-
Our Proposed Model	**78.43**	**98.20**	**98.80**

## Data Availability

Not applicable.

## References

[1] Piza E.L., Welsh B.C., Farrington D.P., Thomas A.L. (2019). CCTV surveillance for crime prevention: A 40-year systematic review with meta-analysis. Criminol. Public Policy.

[2] Suarez J.J.P., Naval P.C. (2020). A Survey on Deep Learning Techniques for Video Anomaly Detection. arXiv.

[3] Morais R., Le V., Tran T., Saha B., Mansour M., Venkatesh S. Learning regularity in skeleton trajectories for anomaly detection in videos. Proceedings of the IEEE Conference on Computer Vision and Pattern Recognition.

[4] Dos Santos F.P., Ribeiro L.S., Ponti M.A. (2019). Generalization of feature embeddings transferred from different video anomaly detection domains. J. Vis. Commun. Image Represent..

[5] Fan Y., Wen G., Li D., Qiu S., Levine M.D., Xiao F. (2020). Video anomaly detection and localization via Gaussian mixture fully convolutional variational autoencoder. Comput. Vis. Image Underst..

[6] Lu C., Shi J., Jia J. Abnormal event detection at 150 fps in matlab. Proceedings of the IEEE International Conference on Computer Vision.

[7] Mehran R., Oyama A., Shah M. Abnormal crowd behavior detection using social force model. Proceedings of the 2009 IEEE Conference on Computer Vision and Pattern Recognition.

[8] Ionescu R.T., Khan F.S., Georgescu M.-I., Shao L. Object-centric auto-encoders and dummy anomalies for abnormal event detection in video. Proceedings of the IEEE Conference on Computer Vision and Pattern Recognition.

[9] Hinami R., Mei T., Satoh S.I. Joint detection and recounting of abnormal events by learning deep generic knowledge. Proceedings of the IEEE International Conference on Computer Vision.

[10] Yan S., Smith J.S., Lu W., Zhang B. (2018). Abnormal event detection from videos using a two-stream recurrent variational autoencoder. IEEE Trans. Cogn. Dev. Syst..

[11] Zhong J.-X., Li N., Kong W., Liu S., Li T.H., Li G. Graph convolutional label noise cleaner: Train a plug-and-play action classifier for anomaly detection. Proceedings of the IEEE Conference on Computer Vision and Pattern Recognition.

[12] Sultani W., Chen C., Shah M. "Real-world anomaly detection in surveillance videos. Proceedings of the IEEE Conference on Computer Vision and Pattern Recognition.

[13] Zhu Y., Newsam S. (2019). Motion-aware feature for improved video anomaly detection. arXiv.

[14] Liu W., Luo W., Lian D., Gao S. Future frame prediction for anomaly detection—A new baseline. Proceedings of the IEEE Conference on Computer Vision and Pattern Recognition.

[15] Sun J., Wang X., Xiong N., Shao J. (2018). Learning sparse representation with variational auto-encoder for anomaly detection. IEEE Access.

[16] Ullah F.U.M., Ullah A., Muhammad K., Haq I.U., Baik S.W. (2019). Violence detection using spatiotemporal features with 3D convolutional neural network. Sensors.

[17] Khan S.U., Haq I.U., Rho S., Baik S.W., Lee M.Y. (2019). Cover the violence: A novel Deep-Learning-Based approach towards violence-detection in movies. Appl. Sci..

[18] Luo W., Liu W., Gao S. Remembering history with convolutional lstm for anomaly detection. Proceedings of the 2017 IEEE International Conference on Multimedia and Expo. (ICME).

[19] Luo W., Liu W., Gao S. A revisit of sparse coding based anomaly detection in stacked rnn framework. Proceedings of the IEEE International Conference on Computer Vision.

[20] Li W., Mahadevan V., Vasconcelos N. (2013). Anomaly detection and localization in crowded scenes. IEEE Trans. Pattern Anal. Mach. Intell..

[21] Wu S., Moore B.E., Shah M. Chaotic invariants of lagrangian particle trajectories for anomaly detection in crowded scenes. Proceedings of the 2010 IEEE Computer Society Conference on Computer Vision and Pattern Recognition.

[22] Tung F., Zelek J.S., Clausi D.A. (2011). Goal-based trajectory analysis for unusual behaviour detection in intelligent surveillance. Image Vis. Comput..

[23] Dalal N., Triggs B. Histograms of oriented gradients for human detection. Proceedings of the 2005 IEEE Computer Society Conference on Computer Vision and Pattern Recognition (CVPR’05).

[24] Dalal N., Triggs B., Schmid C. Human detection using oriented histograms of flow and appearance. Proceedings of the European Conference on Computer Vision.

[25] Zhang D., Gatica-Perez D., Bengio S., McCowan I. Semi-supervised adapted hmms for unusual event detection. Proceedings of the 2005 IEEE Computer Society Conference on Computer Vision and Pattern Recognition (CVPR’05).

[26] Kim J., Grauman K. Observe locally, infer globally: A space-time MRF for detecting abnormal activities with incremental updates. Proceedings of the 2009 IEEE Conference on Computer Vision and Pattern Recognition.

[27] Adam A., Rivlin E., Shimshoni I., Reinitz D. (2008). Robust real-time unusual event detection using multiple fixed-location monitors. IEEE Trans. Pattern Anal. Mach. Intell..

[28] Mahadevan V., Li W., Bhalodia V., Vasconcelos N. Anomaly detection in crowded scenes. Proceedings of the 2010 IEEE Computer Society Conference on Computer Vision and Pattern Recognition.

[29] Cong Y., Yuan J., Liu J. Sparse reconstruction cost for abnormal event detection. Proceedings of the CVPR 2011.

[30] Zhao B., Fei-Fei L., Xing E.P. Online detection of unusual events in videos via dynamic sparse coding. Proceedings of the CVPR 2011.

[31] Hussain T., Muhammad K., Ullah A., Del Ser J., Gandomi A.H., Sajjad M., Baik S.W., de Albuquerque V.H.C. (2020). Multi-View Summarization and Activity Recognition Meet Edge Computing in IoT Environments. IEEE Internet Things J..

[32] Ul Haq I., Ullah A., Muhammad K., Lee M.Y., Baik S.W. (2019). Personalized movie summarization using deep cnn-assisted facial expression recognition. Complexity.

[33] Kwon S. (2020). A CNN-assisted enhanced audio signal processing for speech emotion recognition. Sensors.

[34] Khan N., Ullah F.U.M., Ullah A., Lee M.Y., Baik S.W. (2020). Batteries State of Health Estimation via Efficient Neural Networks with Multiple Channel Charging Profiles. IEEE Access.

[35] Parab A., Nikam A., Mogaveera P., Save A. A New Approach to Detect Anomalous Behaviour in ATMs. Proceedings of the 2020 6th International Conference on Advanced Computing and Communication Systems (ICACCS).

[36] Ullah W., Ullah A., Haq I.U., Muhammad K., Sajjad M., Baik S.W. (2020). CNN features with bi-directional LSTM for real-time anomaly detection in surveillance networks. Multimed. Tools Appl..

[37] Hasan M., Choi J., Neumann J., Roy-Chowdhury A.K., Davis L.S. Learning temporal regularity in video sequences. Proceedings of the IEEE Conference on Computer Vision and Pattern Recognition.

[38] Sabokrou M., Khalooei M., Fathy M., Adeli E. Adversarially learned one-class classifier for novelty detection. Proceedings of the IEEE Conference on Computer Vision and Pattern Recognition.

[39] Zhao Y., Deng B., Shen C., Liu Y., Lu H., Hua X.-S. Spatio-temporal autoencoder for video anomaly detection. Proceedings of the 25th ACM International Conference on Multimedia.

[40] Chang Y., Tu Z., Xie W., Yuan J. Clustering Driven Deep Autoencoder for Video Anomaly Detection. Proceedings of the European Conference on Computer Vision.

[41] Ullah A., Muhammad K., Haydarov K., Haq I.U., Lee M., Baik S.W. One-Shot Learning for Surveillance Anomaly Recognition using Siamese 3D CNN. Proceedings of the 2020 International Joint Conference on Neural Networks (IJCNN).

[42] Tran D., Bourdev L., Fergus R., Torresani L., Paluri M. Learning spatiotemporal features with 3d convolutional networks. Proceedings of the IEEE International Conference on Computer Vision.

[43] Babenko B. (2018). Multiple instance learning: Algorithms and applications. View Artic. PubMed NCBI Google Scholar.

[44] Tomar D., Agarwal S. (2017). Multiple Instance Learning Based on Twin Support Vector Machine. Advances in Computer and Computational Sciences.

[45] Tan K., Hou Z., Ma D., Chen Y., Du Q. (2019). Anomaly detection in hyperspectral imagery based on low-rank representation incorporating a spatial constraint. Remote Sens..

[46] He C., Shao J., Sun J. (2018). An anomaly-introduced learning method for abnormal event detection. Multimed. Tools Appl..

[47] Biradar K., Dube S., Vipparthi S.K. DEARESt: Deep Convolutional Aberrant Behavior Detection in Real-world Scenarios. Proceedings of the 2018 IEEE 13th International Conference on Industrial and Information Systems (ICIIS).

[48] Howard A.G., Zhu M., Chen B., Kalenichenko D., Wang W., Weyand T., Andreetto M., Hartwig A. (2017). Mobilenets: Efficient convolutional neural networks for mobile vision applications. arxiv.

[49] Sandler M., Howard A., Zhu M., Zhmoginov A., Chen L.-C. Mobilenetv2: Inverted residuals and linear bottlenecks. Proceedings of the IEEE Conference on Computer Vision and Pattern Recognition.

[50] Hochreiter S., Schmidhuber J. (1997). Long short-term memory. Neural Comput..

[51] He K., Zhang X., Ren S., Sun J. Deep residual learning for image recognition. Proceedings of the IEEE Conference on Computer Vision and Pattern Recognition.

[52] Kim J., El-Khamy M., Lee J. (2017). Residual LSTM: Design of a deep recurrent architecture for distant speech recognition. arXiv.

[53] Ma J., Tang H., Zheng W.-L., Lu B.-L. Emotion recognition using multimodal residual LSTM network. Proceedings of the 27th ACM International Conference on Multimedia.

[54] Ba J.L., Kiros J.R., Hinton G.E. (2016). Layer normalization. arXiv.

[55] Li X., Zhou Z., Chen L., Gao L. (2019). Residual attention-based LSTM for video captioning. World Wide Web.

[56] (2018). UCF-Crime Dataset. https://www.crcv.ucf.edu/projects/real-world/,.

[57] Raghavendra R., Bue A., Cristani M. Unusual Crowd Activity Dataset of University of Minnesota; 2006. http://mha.cs.umn.edu/proj_events.shtml.

[58] (2013). Avenue Dataset for Abnormal Event Detection. http://www.cse.cuhk.edu.hk/leojia/projects/detectabnormal/dataset.html.

[59] Dubey S., Boragule A., Jeon M. 3D ResNet with Ranking Loss Function for Abnormal Activity Detection in Videos. Proceedings of the 2019 International Conference on Control, Automation and Information Sciences (ICCAIS).

[60] Simonyan K., Zisserman A. (2014). Very deep convolutional networks for large-scale image recognition. arXiv.

[61] Ilg E., Mayer N., Saikia T., Keuper M., Dosovitskiy A., Brox T. Flownet 2.0: Evolution of optical flow estimation with deep networks. Proceedings of the IEEE Conference on Computer Vision and Pattern Recognition.

[62] Bansod S., Nandedkar A. (2019). Transfer learning for video anomaly detection. J. Intell. Fuzzy Syst..

[63] Al-Dhamari A., Sudirman R., Mahmood N.H. (2020). Transfer deep learning along with binary support vector machine for abnormal behavior detection. IEEE Access.

[64] Cheoi K.J. (2020). Temporal Saliency-Based Suspicious Behavior Pattern Detection. Appl. Sci..

